# Putting the Fe into Female Athletes: Insights into Heightened Iron Status and Women’s Australian Football Performance—A Case Study

**DOI:** 10.3390/sports13050136

**Published:** 2025-04-29

**Authors:** Michael Pengelly, Kate Pumpa, David Bruce Pyne, Naroa Etxebarria

**Affiliations:** 1 Research Institute for Sport and Exercise (UCRISE), University of Canberra, Canberra, ACT 2617, Australia; kate.pumpa@ucd.ie (K.P.); david.pyne@canberra.edu.au (D.B.P.); naroa.etxebarria@canberra.edu.au (N.E.); 2 Institute for Sport and Health, University College Dublin, Belfield, 4 Dublin, Ireland

**Keywords:** women, mineral deficiency, training, strength, power, performance

## Abstract

Background: Iron deficiency affects up to 70% of female athletes, yet the effectiveness of improving iron status in team sport athletes remains unclear. Purpose: To evaluate the impact of variations in iron status on physical performance in elite female Australian Rules Football players. Methods: Iron status was measured in 30 players (age 23 ± 4 y; body mass 70 ± 6 kg) across three time-points of a 24-week season. Players identified as iron deficient non-anemic stage 2 in week 1 received an iron infusion. Remaining players were categorized into heightened iron status (FeUP; iron deficient non-anemic stage 1 players who were advised to take iron supplementation; *n* = 8) or non-supplemented (Ctrl; week 10: *n* = 17; week 24: *n* = 16) groups following week 10. Relative external load measures (e.g., total distance at different velocities), and strength and power measures (e.g., counter-movement jump, reactive strength index) were recorded in weeks 10 and 24 (same weeks as blood samples) to compare physical performance between FeUP and Ctrl groups. Results: Iron status improved in FeUP (30 µg/L to 49 µg/L) and reduced in Ctrl (39 µg/L to 34 µg/L) between weeks 10 and 24. Iron deficiency prevalence among all players was 47–54%. FeUp exhibited small to moderate improvements (5–19%) in some external load measures but no consistent improvement across all measures. At week 24, FeUP showed trivial to moderate differences outperforming Ctrl in seven physical performance measures (e.g., both hip adduction tests), but Ctrl outperformed FeUP in all other measures. Conclusion: Heightened iron status does not consistently enhance physical performance, although avoiding deficiency remains essential for athlete health.

## 1. Introduction

Iron is an important mineral that has marked effects on various exogenous and endogenous factors underpinning sports performance [1,2,3]. The focus on iron’s role in sports performance has principally concerned aerobic-dominant athletes, including rowers [1,2], cross-country skiers [4], and runners [5,6], assessed via time-trial performance ranging between 8 and 18 min [1,2,5,6] and maximal aerobic assessment [7,8]. However, these protocols do not typically characterize intermittent team sports performance, making it difficult to examine the relationships between micronutrient status and the physical capacities of team sport athletes. Nevertheless, the mechanisms by which iron supports successful aerobic-dominant sports performance, such as oxidative phosphorylation of adenosine di-phosphate to adenosine tri-phosphate, oxygen transportation and uptake, and erythropoiesis [2,9,10,11], apply equally to intermittent team sports athletes. Therefore, iron deficiency may impair physical capacities in intermittent team sports that are reliant on oxygen delivery to working skeletal muscle (for endurance) [2,5,12], and tissue oxygenation for repeated high-intensity intervals, strength and power training, and recovery [12,13].

The prevalence of iron deficiency is greater in female athletes [14,15,16], driven by a number sex-specific factors (such as greater prevalence of vegetarian and vegan athletes, menstruation). The sex-specific factors in which female athletes lose iron stores are in addition to several mechanisms in which athletes of both sexes are already predisposed to greater iron losses (e.g., gastrointestinal bleeding, environmental factors, iron regulatory hormones) [17,18,19]. To account for these factors, the recommended dietary intake for females (18 mg/day) is higher than males (8 mg/day). However, female athletes may still be predisposed to substantial iron losses even when satisfying these recommendations following periods of intensified training [17], suggesting the current recommended dietary intake may be inadequate in some individuals to support training and competition performance. This inadequacy has been highlighted in research by Mielgo-Ayuso et al. [20], who reported that 30% of female volleyball players developed pre-latent iron deficiency (sFer 20–60 µg/L) after 11 weeks of training and competition, despite consuming approximately 26 mg/day of dietary iron. Improving the iron status of iron deficient (ID) female athletes may therefore be necessary to maintain adequate sFer levels and avoid associated performance deficits. Understanding the effects of heightened iron status on physical performance measures underpinning sports performance will inform player medical, nutrition and training management.

Studies on the benefits of heightened iron status in elite ID female athletes are limited, particularly for intermittent team sport athletes [7,13]. Australian Football League Women’s (AFLW) is typified by a range of physical capacities including endurance, strength, power, and intermittent short, high-intensity efforts [21]. Each of these physical capacities is underpinned by mechanisms requiring adequate iron levels. In AFLW players, it appears iron is key in sustaining higher work rates aerobically, as well as tissue oxygenation and clearance of by-products between repeated efforts [21]. Specifically, the iron requirement in AFLW players appears vital for actions including wrestling, tackling, jostling, and jumping to out mark an opponent—actions typified by high-levels of strength and power. Further, iron appears important in enabling players to cover high total distances at high velocities. Therefore, the aim of this study was to evaluate the impact of variations in iron status on physical performance measures in AFLW athletes.

## 2. Materials and Methods

### 2.1. Experimental Design and Subjects

This study followed a 24-week prospective longitudinal design during the 2024 AFLW season to evaluate the benefit of monitoring changes in the iron status of ID female athletes, and associations with changes in the physical performance of AFLW players. The AFLW is the premier women’s Australian Football competition in Australia. Participants were recruited from a single AFLW team using convenience sampling. Thirty players (age 23 ± 4 y; height: 171 ± 7 cm; body mass 70 ± 6 kg; mean ± SD) competing in the AFLW from the same team participated, representing all playing positions (defenders: *n* = 5; midfielders: *n* = 13; forwards: *n* = 6; key position (e.g., ruck, center half forward): *n* = 6). The team competed in 12 matches across the season between August and November 2024 (weeks 11–23). The Home and Away season included one game following a 4-, 6-, 8-, and 9-day break, and three games following either a 5- or 7-day break. The team also competed in one finals game coming off a 7-day break. All training days included a ~70 to 120 min on-field training session and a ~45 to 60 min gym session. During the preseason (10 weeks), the team trained 4 days a week, while training days in-season were determined by days between games. That is, the team typically trained once per week on a 4-day break, twice on 5- and 6-day breaks, and three times on breaks of 7 or more days. All players were informed of the study design, risk, and benefits before providing their written informed consent. This study was approved by the University of Canberra Human Research Ethics Committee (Approval number 12213).

### 2.2. Methodology

**Figure 1:**
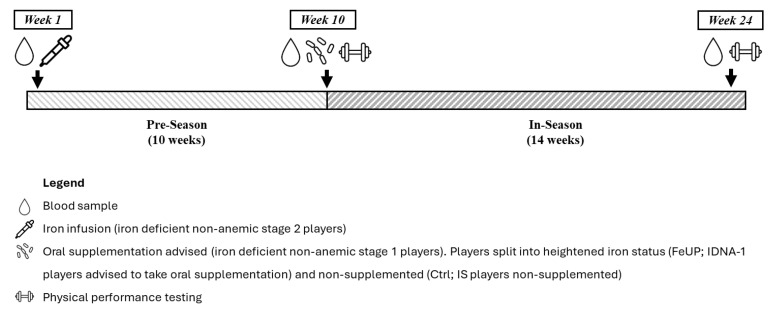
Schematic overview of the methodology employed throughout the 2024 AFLW season.

Blood samples (~10 mL) were collected at three time-points across the entire 2024 AFLW season to assess iron status (start of pre-season [week 1, end of May], end of pre-season [week 10, end of July], and end-season [week 24; November]; Figure 1). Blood samples were collected from the same two trained phlebotomists from an accredited local pathology clinic, and with biomarker analysis conducted at the pathology clinic. A total of 24 players provided all three samples while 5 players had two samples collected. One player withdrew from the study during week 9 for personal reasons, leaving a final sample of 29 players. At the time of blood testing, no players were sick or injured. Samples were collected between 1230 and 1500 h following a minimum of 24 h rest.

In the current study, stage one iron deficiency non-anemia (IDNA-1) was defined as sFer < 40 µg/L and hemoglobin concentration (Hb) > 120 g/L; stage two iron deficiency non-anemia (IDNA-2) sFer < 20 µg/L and Hb > 120 g/L; and stage three iron deficiency anemia (IDA-3) sFer < 12 µg/L, Hb < 120 g/L, mean cellular volume (MCV) > 80 fl, mean cell hemoglobin > 28 pg [20,21]. Functional iron-deficiency was defined as a sFer between 40 to < 100 µg/L and iron normal as a sFer > 100 µg/L [13,22].

Two players were identified as IDNA-2 in week 1, and received an iron infusion (10 mL, 500 mg elemental iron) from the team’s sports physician, with no other players identified as IDNA-2 for the remainder of the study. Players with IDNA-1 in week 10 (FeUP; *n* = 8) were advised to take oral iron supplementation (Maltofer: 370 mg iron polymaltose; ~100 mg elemental iron bi-daily) under the guidance of the team’s sports dietitian for the remainder of the season. All non-supplemented players (iron sufficient) formed the control group (Ctrl; week 10: *n* = 17; week 24: *n* = 16).

For the purpose of this study, physical performance was used as an encompassing term referring to both external load (GPS) and strength and power measure. Players wore a Catapult Vector S7 GPS unit (Catapult Sports, Melbourne, Australia) during training and matches to measure external load physical performance. The validity of these devices for reporting external load variables has been established in previous research [23]. GPS units were positioned between the scapulae in a player’s guernsey, with data downloaded after each session. External game and training load variables collected included total distance (TD), PlayerLoad^TM^, and distances covered at various velocities (TD > 50%, TD > 70%, TD > 80%, and TD > 90%) relative to each player’s maximal velocity. External load measures were standardized relative to each hour of game/training to cater to differences in the number and duration of weekly sessions per athlete. Players who completed modified training during weeks 1, 10, or 24 were excluded from analysis.

Weekly screening assessments, including hip adduction, hip abduction, reactive strength index (RSI), counter-movement jump (CMJ), isometric 30˚ hamstring hold, and Nordic hamstring curl were used to monitor changes in strength and power. CMJ and RSI data were collected using ForceDecks (Vald Performance, Newstead, QLD, Australia), hip adduction and abduction data collected using the ForceFrame (Vald Performance, Newstead, QLD, Australia), and isometric 30° hamstring hold and Nordic hamstring curl data was collected using the Nordbord (Vald Performance, Newstead, QLD, Australia). These tests are valid assessments of physical capacities [24,25] and are in accordance with routine practice in Australian Football [26,27,28], as well as routine assessments implemented by the team’s high-performance and medical staff. All assessments except reactive strength index were standardized relative to each player’s body weight.

### 2.3. Statistical Analysis

Descriptive statistics and seasonal differences in iron markers between FeUP and Ctrl groups are presented as mean ± SD. A Mann–Whitney U test was used to analyze inter-group difference in all performance measures between FeUP and Ctrl players for weeks 10 and 24. The Wilcoxon Signed Rank test was used to analyze intra-group differences in all performance measures for FeUP players between weeks 10 and 24. Effect sizes with a 95% confidence interval (CI) were calculated to quantify inter- and intra-group differences in performance, with magnitudes interpreted as follows: <0.20 (trivial), 0.20 to 0.59 (small), 0.60 to 1.19 (moderate), 1.20 to 1.99 (large), and >2.00 (very large).

A linear mixed-effects model was used to analyze the effect of sFer on each performance measure. For the linear–mixed effects modeling, the two players who received an iron infusion in week 1 were included in the FeUP group. Fixed effects included sFer, group (FeUP or Ctrl), and the week, with the athlete included as a random effect. For all external load measures, position was also included as a fixed effect. The models were fitted using the ‘lmer’ function from the ‘lme4′ package employing the restricted maximum likelihood (REML) estimation method in RStudio version 4.3.1. The model for external load measures was specified as follows: lmer (Response ~ 1 + sFer × Position + sFer × Week + sFer × Group + (1|Athlete), data = data_source). The model for strength and power measures was specified as follows: lmer (Response ~ 1 + sFer × Group + sFer × Week + (1|Athlete), data = data_source). Graphics were developed with the ggplot2 package. Estimated marginal means were fitted with the ‘emmip’ function utilizing 95% CIs. Pearson’s correlation values were interpreted as <0.20 (very weak), 0.20–0.39 (weak), 0.40–0.59 (moderate), 0.60–0.79 (strong), and >0.80 (very strong). Goodness of fit was assessed through residual analysis, with statistical significance set at *p* < 0.05.

## 3. Results

### 3.1. Seasonal Iron Status

**Figure 2:**
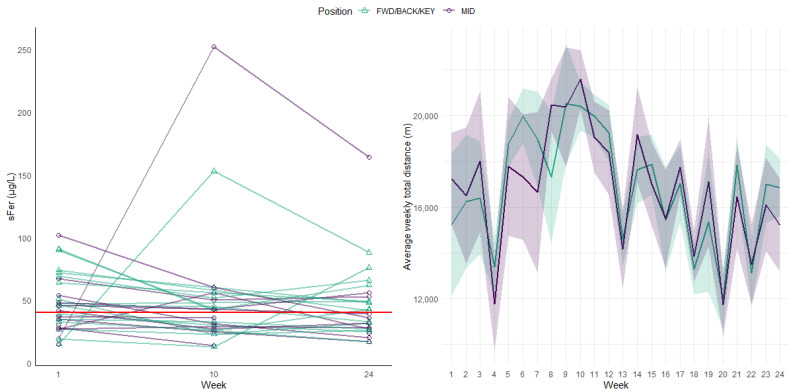
Seasonal variation in serum ferritin (sFer) (left) and weekly training loads (right) throughout the 2024 AFLW season for all players. All players were classified as either a forward, back or key position players (FWD/BACK/KEY; green triangles) or a midfielder (MID; purple circle). The red line indicates an sFer of 40 µg/L, the diagnostic cut off used in the current study to categorize iron deficiency.

All players (*n* = 30) provided a venous blood sample in week 1, with participation decreasing throughout the season due to player unavailability (e.g., injury, illness, personal reasons; week 10: *n* = 27; week 24: *n* = 26). The mean ± SD values for all iron indices of both infusion players are provided in Table 1. The sFer status for both players receiving an infusion increased more than ten-fold by week 10 following the infusion in week 1. However, sFer status then decreased by ~40% in both players between week 10 and 24. All iron indices for FeUP and Ctrl (mean ± SD) are provided in Table 2, and an overview of the team’s weekly external training loads and seasonal fluctuations in iron status for each player are presented in Figure 2. Mean weekly training loads ranged between ~12,000 and ~21,000 m throughout the season, while ~70% of all samples ranged between 9 and 60 µg/L. The mean sFer status declined from 47 µg/L to 39 µg/L (excluding the sFer status of the two players who received an iron infusion following week 1) with the prevalence of iron deficiency in the team increasing from 47% in week 1 to 54% in week 24. In the Ctrl group, sFer levels decreased by 13% between weeks 10 and 24, whereas the FeUP group exhibited a 63% increase. There were generally moderate differences in iron indices with higher concentration of biomarkers for Ctrl at week 10, but higher concentrations for FeUP at week 24.

### 3.2. Iron Infusion and Physical Performance

A variable response in physical performance was identified in two players who received iron infusions. External load measures ranged from a 24% reduction to a 180% improvement, with both players generally demonstrating reductions in total distance covered at all velocity thresholds throughout the season (in both training and competition) compared to week 1 (Table 1). RSI improved by 3–32% in one player (Player 1) who received an iron infusion. Strength and power performance measures generally declined for both players after week 1, despite both recording their lowest sFer levels at that time.

### 3.3. Heightened Iron Status and Physical Performance

**Figure 3:**
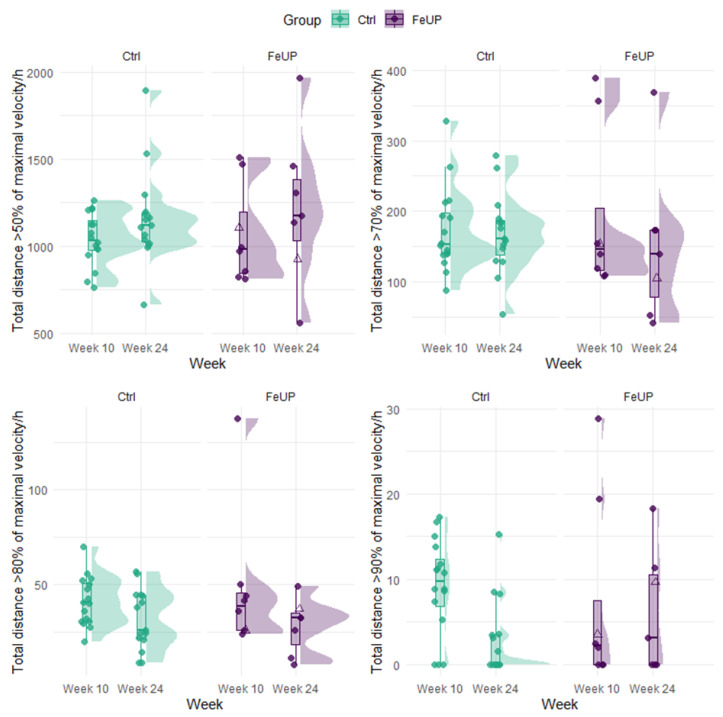
Distribution plot of the relative total distance covered at each velocity per hour for each player separated by group (Ctrl and FeUP) and week. The FeUP group (purple) represents those players with heightened iron status between weeks 10 and 24. Unfilled purple triangles indicate the two players who received an iron infusion after week 1, while filled purple circles represent all other FeUP players. The Ctrl group (green) represents all non-supplemented players between weeks 10 and 24.

**Figure 4:**
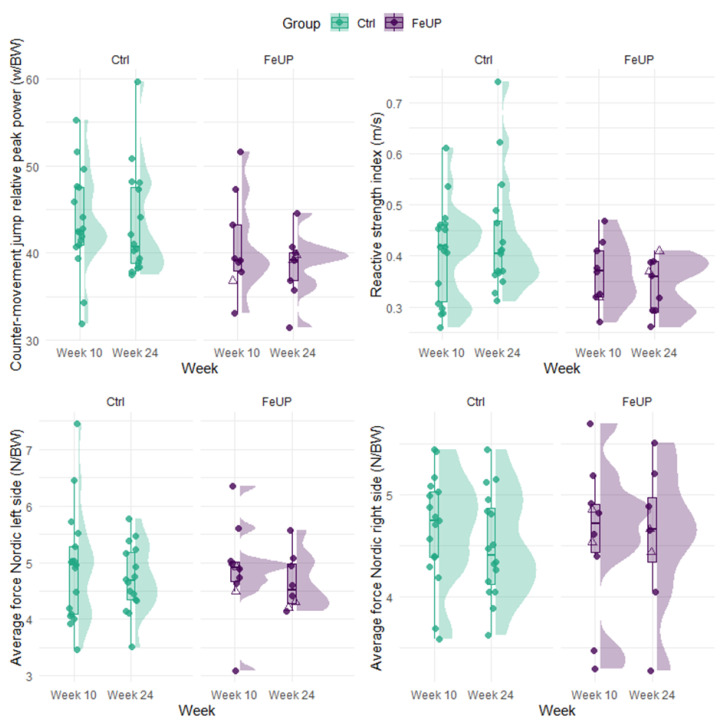
Distribution plot of the relative counter-movement jump (CMJ) relative power, reactive strength index, and Nordic hamstring curl force output for each player separated by group (Ctrl and FeUP) and week. The FeUP group (purple) represents those players with heightened iron status between weeks 10 and 24. Unfilled purple triangles indicate the two players who received an iron infusion after week 1, while filled purple circles represent all other FeUP players. The Ctrl group (green) represents all non-supplemented players between weeks 10 and 24.

Changes in the direction and magnitude of performance among FeUP and Ctrl groups on external load measures were also inconsistent between weeks 10 and 24. The running metrics of TD, TD > 50%+, and PlayerLoad improved by 7–19% for FeUP and 10–12% for Ctrl, but, in contrast, high-speed running (> 70%+) was reduced for both groups (trivial to moderate effect; Table 3, Figure 3, and Online Supplementary File 1: Figure S1). Most strength and power measures (*n* = 7) declined or showed no change for both groups between weeks 10 and 24 (Table 3, Figure 4, and Online Supplementary File Figure S2). There were improvements in hip abduction left side, and hip adduction left and right side by 2–9% between weeks 10 and 24. In week 24, the Ctrl players outperformed FeUP players in the RSI. Similarly, differences in CMJ relative power output in week 24 moderately favored Ctrl, though this finding was not statistically significant. Trivial to small differences of −7 to 8% were evident between both Nordic hamstring curls, hip abduction and adduction, and isometric 30˚ hamstring holds for both intra- and intergroup comparisons (Table 3 and Online Supplementary File S1: Table S1).

**Figure 5:**
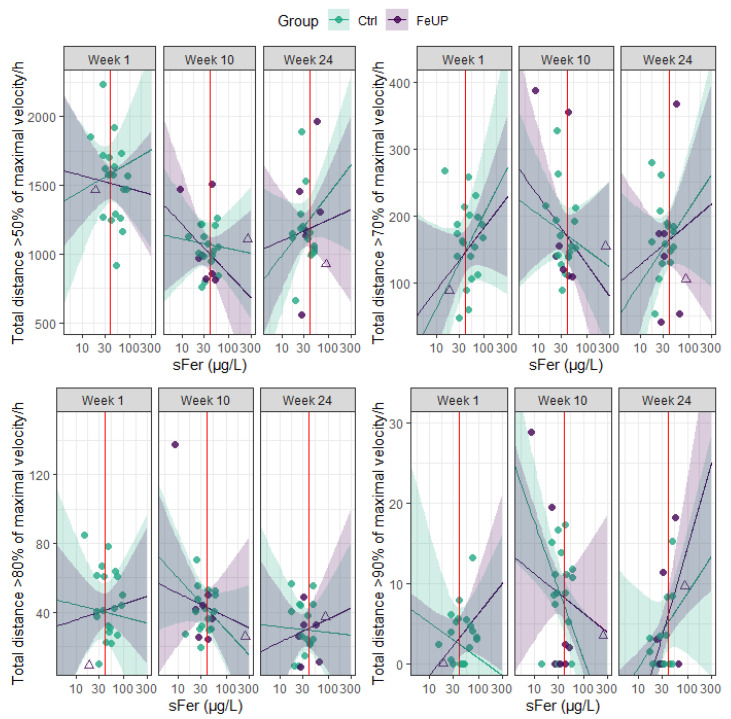
Scatterplot of the relative total distance covered at each velocity per hour for each individual athlete plotted against serum ferritin (sFer), separated by week. The purple line represents the predicted total distance and PlayerLoad covered by the players with heightened iron status between weeks 10 and 24 (FeUP) per hour, with 95% CIs in purple. Unfilled purple triangles indicate the two players who received an iron infusion after week 1, while filled purple circles represent all other FeUP players. The green line represents the predicted total distance and PlayerLoad covered by the non-supplemented players (Ctrl) per hour, with 95% CIs in green. The red line indicates a threshold value for sFer of 40 µg/L, the diagnostic cut off used in the current study to categorize iron deficiency.

**Figure 6:**
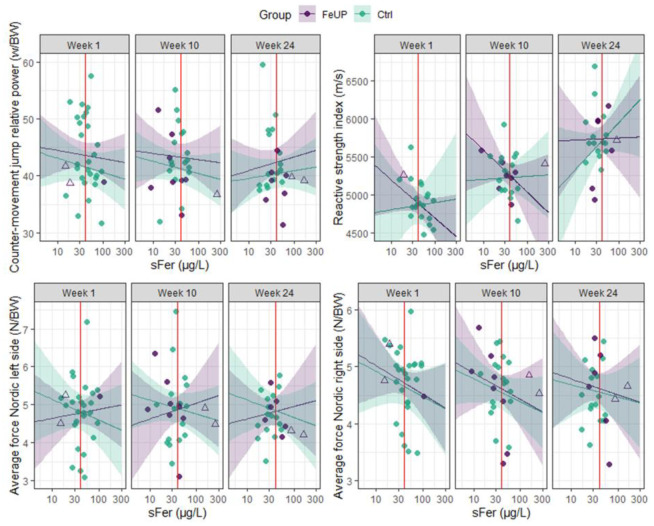
Scatterplot of the relative counter-movement jump relative power, reactive strength index, and Nordic hamstring curl force output for each individual athlete plotted against serum ferritin (sFer), separated by week. The purple line represents the predicted total distance and PlayerLoad covered by the players with heightened iron status between weeks 10 and 24 (FeUP) per hour, with 95% CIs in purple. Unfilled purple triangles indicate the two players who received an iron infusion after week 1, while filled purple circles represent all other FeUP players. The green line represents the predicted total distance and PlayerLoad covered by the non-supplemented players (Ctrl) per hour, with 95% CIs in green. The red line indicates a threshold value for sFer of 40 µg/L, the diagnostic cut off used in the current study to categorize iron deficiency.

The associations between iron status and performance are visualized in Figure 5 and Figure 6, and ‘Online Supplementary File S1: Figure S1 and S2. Marginal r-squared values for all performance measures ranged between 0.0 and 0.6, indicating very weak to strong correlations between each performance measure and the fixed effects (sFer, position, and week) (Table 4, and ‘Online Supplementary File S1: Tables S2–S5). Conditional r-squared values increased to 0.3 to 0.9 indicating weak to very strong associations when accounting for the random effect (athlete) as well as the fixed effects (Table 4, and ‘Online Supplementary File S1: Tables S2–S5).

## 4. Discussion

There was a highly variable pattern of changes in measures of iron status and physical performance across a full 24-week season in AFLW players. Specifically, the effect of improved iron status on the physical performance of AFLW players ranged between −47% to +19%. Like other intermittent team sports, some moderating variables (e.g., playing position, change in weekly training loads) appear to influence physical performance more, modifying the impact of improved iron status on performance. The study is constrained by the absence of supplementation compliance recording in practice and highlights the necessity for teams and organizations to implement standardized iron monitoring and supplementation protocols to support overall athlete health and performance given the high prevalence in iron deficiency observed.

The prevalence of iron deficiency in the team was 47% at the start of the season (week 1), increasing to 54% by the end of the season (week 24). The higher prevalence of iron deficiency at the end of the season reflects the deteriorating mean sFer status of the team which reduced from 47 µg/L to 39 µg/L between weeks 1 and 24. Without iron interventions, the prevalence of iron deficiency in weeks 10 and 24 would likely have been higher. It is possible that the sFer status of the team may have declined without the two IDNA-2 players receiving an infusion following week 1, and eight players consuming oral supplementation either by their own discretion or following advice from the team’s sports dietitian following week 10. Importantly, 70% of the team were ID at least once throughout the season, which is a higher incidence than that reported in runners and triathletes [16], soccer players [14], and rugby players [15], but is similar to track and field athletes [29] and ballet dancers [30]. Further, 100% of the team had functional iron deficiency (<100 µg/L) at least once throughout the season, highlighting the necessity for teams and organizations to have structured protocols in place that support athletes.

Despite a heightened iron deficiency prevalence in team sport athletes [14] comparable to aerobic-dominant athletes [16], little research has addressed performance measures reflective of intermittent team sports. Studies on the effects of heightened iron status in team sport athletes have focused primarily on volleyball [13] and netball [7] players, and endurance athletes [12], with mixed findings similar to the current study. For example, while only four volleyball players were categorized as clinically ID (sFer: <30 µg/L; Hb: >120 g/L), with three receiving iron supplementation (325 mg/d ferrous sulfate; ~105 mg/d elemental iron), strength and power performance (7–10 repetition maximal) in the iron supplementation group improved by 26 to 45% compared to the control group (−6 to +10%). Notably, the control group experienced marked reduction in hematological biomarkers (sFer, Hb, ferritin, transferrin saturation index: −35% to −8%). In contrast, Blee et al. [7] observed no change in maximal aerobic capacity assessed via a multi-stage 20 m shuttle-run test and a 3% reduction in average power via a 5 × 6 s sprint cycle ergometer test. In the current study, while sFer increased more than ten-fold for both players receiving an infusion, the response across all performance measures were highly variable with a similar result exhibited by FeUP between weeks 10 and 24.

Other moderating variables (i.e., playing position and change in weekly training loads) tend to have a greater, albeit expected, impact on external load performance than increased iron status. Specifically, midfielders tend to have an initial 2 to ~160 times greater effect on external load performance than a one microgram increment in sFer. Importantly, both FeUP and Ctrl groups consisted of ~50% midfielders. However, grouping the defenders, forwards, and key position players together may obscure position-specific performance discrepancies, especially given the small sample size. Studies show that half-back and half-forward lines (in Australian Football) cover approximately 30% more total distance during a match, and 20% more moderate-speed distance (10–15 km/h) per minute than full-back and full-forward lines [21]. Larger studies that compare heightened iron status against controls, and those stratified by position, would provide a clearer understanding of how iron status affects external game and training load performance.

While position tends to affect external load performance more than sFer status, the change in planned weekly loads had an approximate 3 to 7 times greater effect than playing position. The volatility of weekly load changes is evident in Player 1 s performance following the iron infusion. That is, the increase in TD > 70%+ and TD > 80%+ between weeks 1 and 10 may initially be attributed to the heightened sFer status. However, despite an 863% increase in sFer, TD > 70%+ decreased by −64% between weeks 1 and 24. Based on the ReML analysis, TD > 70%+ was expected to improve by ~250% by week 24. The effect of moderating variables on AFLW external load performance mirrors that of other intermittent team sport athletes with position and weekly loads having 7 to 329 times greater impact on performance than sFer in elite female rugby league players [31].

The greater influence of position and weekly loads does not diminish the potential benefits of heightened iron status on external load performance, despite the inconsistent responses observed in the two players who received an iron infusion. For example, a player with an sFer of 100 µg/L is predicted to outperform an ID athlete with an sFer of 10 µg/L by approximately 50–200% in high-speed running (TD > 70%+) distance covered per hour. It would be beneficial to further explore performance measures that minimize the effect of moderating variables in environments with recommended iron supplementation protocols. This information would provide a clearer understanding of how intermittent team sport athletes might benefit from heightened iron status.

Similarly to the assessed external load measures, strength and power performance may also be affected more by moderating variables, including change in weekly training loads, and a learning effect in which athletes become more accustomed to the physical assessment employed. For example, one player (Player 1) who received an iron infusion showed a 3% improvement in RSI between weeks 1 and 10, followed by a further 28% between weeks 10 and 24. The initial 3% improvement in RSI is misrepresented by the 1300% increase in sFer, given that the largest RSI improvement occurred between weeks 10 and 24 (+28%), when sFer decreased by 35%. It is likely the improvement is more attributable to a learning effect rather than the direct influence of sFer. It is also important to consider that Player 1 was a first-year rookie (18 years old), and her training age was likely still immature. For example, CMJ relative power reduced by 5% during the period of greatest increase in sFer, but experienced a 7% improvement between weeks 10 and 24 when sFer declined. It appears that improvements in RSI and CMJ relative power were likely due to a learning effect rather than changes in iron status following the iron infusion.

The observed changes in weekly load for strength and power performance are a greater reflection of strength monitoring and potentially adaptation, rather than just load variation itself given the assessments employed. Thus, it is unsurprising that weekly load had little effect on strength and power performance, especially when compared to external load performance. However, when evaluating the impact of iron status on strength and power, it is important to also consider the influence of cumulative weekly external training loads on performance. In team sport athletes, it is well documented that measures of fatigue typically increase throughout the season in response to accumulative training loads, consequently affecting lower body strength and power [32,33,34,35]. In the current study, both FeUP and Ctrl groups demonstrated either no change or declines in performance between weeks 10 and 24 for most measures (70%). It is possible that trivial or negative changes in strength and power performance for both groups might be explained by fatigue, as the training loads accumulate throughout the season. It is important to note that wellness questions addressing sleep quality, soreness, readiness, and menstrual cycle severity were recorded daily throughout the season in the current study. However, Likert scale data were heavily skewed towards a single answer for each question (66–82%) and, together with the second most popular response, these answers accounted for 81–97% of all responses (soreness: 85%; readiness: 97%; sleep quality: 81%; menstrual cycle severity: 91%). The limited variability reduced the usefulness of the data thus these measures were not included in the linear models.

Seasonal fatigue likely does not account for the mainly trivial-to-small difference in performance between groups. In theory, if a higher iron status is beneficial for greater strength and power performance, the greater iron status for FeUP at week 24 should have attenuated any effect of fatigue and higher external load compared to Ctrl. However, this was not the case. Instead, the similarity in iron status between both groups may be responsible for the negligible difference between groups. That is, at week 24, there was only a difference of 15 µg/L in sFer between both FeUP and Ctrl, which is unlikely to be substantial enough to warrant meaningful differences in strength and power performance. Further, the proposed mechanism underpinning improvements in strength and power with heightened iron status is enhanced tissue oxygenation, which facilitates recovery between sets and sessions [13,36]. All players were categorized as either IDNA-1 or IDNA-2 indicating the severity of iron deficiency was insufficient to hinder hemoglobin concentration. As such, no substantial difference in performance would be expected between weeks 10 and 24 for any player.

Despite only non-anemic athletes being proposed to experience impaired strength and power performance, clinically ID (sFer < 30 µg/L) female athletes have displayed compromised isokinetic strength performance of up to −23% in 10 of the 12 assessments [12]. In contrast, clinically ID volleyball players improved strength in hand power performance by 26–45% following iron supplementation. Although the findings of Cialdella-Kam et al. [12] and Mielgo-Ayuso et al. [13] were not statistically significant—likely due to small sample sizes—they challenge the assumption that only anemic athletes experience impaired performance. To detect meaningful differences in strength and power performance, stratifying participants by sFer status, similar to position-specific analyses for external load measures, may be more informative in larger studies. However, this approach was not feasible in the present study, as ~80% of blood samples for both FeUP and Ctrl at weeks 10 and 24 were ≤ 50 µg/L, limiting the ability to differentiate performance outcomes based on iron status.

### 4.1. Limitations

Given that this study was conducted in a real world or applied setting, there are inherent limitations, but also significant strengths. Most notably, supplementation compliance was not recorded in this applied setting, making it difficult to discern the impact of iron supplementation on physical performance in AFLW players beyond the observed improvements in iron status. Nevertheless, FeUP sFer status improved by 63% between weeks 10 and 24 (30 µg/L to 49 µg/L), exceeding the magnitude of effects observed in previous research. For example, elite female volleyball players experienced an 8% reduction in sFer over 12 weeks following iron supplementation of ~105 mg/d of elemental iron [13], while collegiate rowers experienced a 12% increase following ~16 mg/d of elemental iron [37]. Secondly, the strength and power assessments employed in the study were commonly utilized measures of limb asymmetries and assessments implemented by the club. It is plausible that sFer may have a more pronounced effect on compound movements, such as the bench press or squat, as shown in elite volleyball players [13]. Incorporating a more comprehensive physical test battery (e.g., inclusive of a Yo-Yo Intermittent Recovery Test, bench press, and squat) may yield useful information in identifying performance changes associated with heightened sFer status. Finally, it would have been beneficial to include dietary intake assessments (e.g., food diaries) and more comprehensive menstruation data. Food diaries were deemed inappropriate for 17 of the 30 players by the team’s sports dietitian. Of the remaining 13 players, compliance was low, with only 23% (*n* = 3) of players completing a food diary. Therefore, data for the food diaries were not included in the results. The inclusion of menstruation data (i.e., heaviness of bleeding) may enhance the strength of future analyses, particularly when interpreting individual responses to supplementation. It would be beneficial for future research to integrate this information to better capture the multifactorial nature of iron metabolism in female athletes.

### 4.2. Practical Implications

Given that 70% of the players were categorized as IDNA-1 or IDNA-2, and 100% of the players presented with sub-optimal iron stores (sFer < 100 µg/L) at least once throughout the season, regular iron assessments are encouraged for intermittent female team sport athletes. Moreover, recommended protocols are necessary to address iron deficiencies and substantial decreases in iron status throughout the season, even in the absence of IDNA-1. While the effect of heightened status on physical performance measures was highly variable in AFLW players, iron entertains a variety of roles facilitating sports performance beyond physical performance (e.g., cognition, collagen synthesis), thereby necessitating that interventions are promptly administered to ID and functionally ID athletes with low sFer status.

## 5. Conclusions

Heightened iron status had an inconsistent effect on physical performance measures in AFLW players. Physical performance measures are more sensitive to moderating variables (playing position, changes in weekly load) than sFer. Given the interpretations are limited by the homogeneity in iron status, seasonal iron monitoring is recommended in team sport athletes to identify larger fluctuations that may impose negative effects on physical performance (e.g., an athlete with an initial sFer > 100 µg/L declining to 50 µg/L).

## Figures and Tables

**Table 1:** Iron indices and physical performance measures recorded throughout the season for two players who received an iron infusion, with differences in each measure expressed as a percentage. A variable pattern of increases and decreases over the 24 weeks was evident in iron measures, running demands, and strength and power.

Performance Measure	Athlete	Week 1	Week 10	Week 24	Difference in Measure (%)		
					Week 1:Week 10	Week 1:Week 24	Week 10:Week 24
**sFer (µg/L)**	Player 1	19	252	164	1326%	863%	−35%
	Player 2	15	153	88	1020%	587%	−42%
**Hb (g/L)**	Player 1	126	138	126	10%	0%	−9%
	Player 2	132	141	132	7%	0%	−6%
**MCV (fl)**	Player 1	92	92	92	0%	0%	0%
	Player 2	94	92	94	−2%	0%	2%
**McHb (pg)**	Player 1	30	30	30	0%	0%	0%
	Player 2	31	32	31	3%	0%	−3%
**TD > 50% (m)**	Player 1	1465	1107	881	−24%	−40%	−20%
	Player 2	1838	-	927	-	−50%	-
**TD > 70% (m)**	Player 1	88	154	31	75%	−64%	−80%
	Player 2	0	-	105	-	-	-
**TD > 80% (m)**	Player 1	9	26	0	180%	−100%	−100%
	Player 2	0	-	37	-	-	-
**TD > 90% (m)**	Player 1	0	4	0	-	-	-
	Player 2	0	-	10	-	-	-
**CMJ relative power (w/BW)**	Player 1	39	37	39	−5%	1%	7%
	Player 2	42	-	39	-	−5%	-
**RSI (m/s)**	Player 1	0.3	0.3	0.4	3%	32%	28%
	Player 2	0.41	-	0.37	-	−10%	-
**Nordic left (N/BW)**	Player 1	5.2	4.5	4.2	−13%	−19%	−7%
	Player 2	4.5	4.9	4.3	9%	−4%	−12%
**Nordic right (N/BW)**	Player 1	5.4	4.5	4.7	−17%	−13%	4%
	Player 2	4.8	4.9	4.4	2%	−8%	−10%

sFer—serum ferritin; Hb—hemoglobin concentration; MCV—Mean cellular volume; MCHb—Mean cellular hemoglobin; TD > 50%—total distance covered greater than 50% of maximal velocity; TD > 70%—total distance covered greater than 70% of maximal velocity; TD > 80%—total distance covered greater than 80% of maximal velocity; TD > 90%—total distance covered greater than 90% of maximal velocity; CMJ—counter-movement jump; RSI—reactive strength index; Nordic left—Nordic hamstring curl left side; Nordic right—Nordic hamstring curl right side.

**Table 2:** Iron indices (mean ± SD) recorded throughout the season for all players, and between week 10 and week 24 for AFLW players with heightened iron status (FeUP), and non-supplemented AFLW players (Ctrl) with effect size comparisons.

Iron Measure	All Players	FeUP	Cohen’s d (90% CIs)	*p* Value	Ctrl	Cohen’s d (90% CIs)	*p* Value
	Mean (SD)	Mean (SD)	FeUP Week 10:Week 24		Mean (SD)	FeUP:IS	
**n=**							
**Week 1**	30						
**Week 10**	25	8			17		
**Week 24**	24	8			16		
**sFer**							
**Week 1**	47 (23)						
**Week 10**	36 (15)	30 (15)			39 (14)	0.6 (−0.1 to 1.4)	0.145
**Week 24**	39 (16)	49 (19)	−1.1 (−1.8 to −0.3)	0.012	34 (12)	−1.1 (−1.8 to −0.3)	0.057
**Hb (g/L)**							
**Week 1**	132 (6)						
**Week 10**	140 (18)	143 (9)			139 (22)	−0.2 (−0.9 to 0.5)	0.058
**Week 24**	132 (6)	134 (6)	1.9 (0.9 to 2.9)	0.012	131 (6)	−0.5 (−1.2 to 0.3)	0.355
**MCV (fl)**							
**Week 1**	92 (3)						
**Week 10**	92 (3)	94 (2)			91 (4)	−1 (−1.7 to −0.2)	0.038
**Week 24**	92 (4)	94 (2)	0.3 (−0.3 to 0.9)	0.339	92 (4)	−0.6 (−1.3 to 0.1)	0.072
**MCHb (pg)**							
**Week 1**	30 (1)						
**Week 10**	31 (1)	31 (1)			30 (1)	−0.6 (−1.4 to 0.1)	0.093
**Week 24**	31 (1)	31 (1)	0 (−0.8 to 0.8)	1.00	30 (1)	−0.6 (−1.4 to 0.1)	0.129

sFer—serum ferritin; Hb—hemoglobin concentration; MCV—Mean cellular volume; MCHb—Mean cellular hemoglobin.

**Table 3:** Physical performance measures (mean ± SD) recorded during week 10 and week 24 between players with heightened iron status (FeUP initial sFer < 40 µg/L) and non-supplemented (Ctrl; initial sFer ≥ 40 µg/L) AFLW players with effect size comparisons.

Iron Measure	FeUP	Cohen’s d (90% CIs)	*p* Value	Ctrl	Cohen’s d (90% CIs)	*p* Value
	Mean (SD)	FeUP Week 10:Week 24		Mean (SD)	IS:FeUP	
**TD > 50% (m)**						
**Week 10**	1061 (300)			1039 (153)	−0.1 (−0.9 to 0.6)	0.671
**Week 24**	1266 (459)	−0.4 (−1 to 0.2)	0.401	1161 (272)	−0.3 (−1.1 to 0.5)	0.302
**TD > 70% (m)**						
**Week 10**	196 (121)			173 (60)	−0.3 (−1 to 0.5)	0.624
**Week 24**	158 (118)	0.7 (0 to 1.3)	0.093	168 (56)	0.1 (−0.7 to 0.9)	0.470
**TD > 80% (m)**						
**Week 10**	51 (39)			41 (13)	−0.4 (−1.2 to 0.3)	0.974
**Week 24**	27 (15)	0.6 (−0.1 to 1.2)	0.093	32 (16)	0.3 (−0.5 to 1.1)	0.622
**TD 90% (m)**						
**Week 10**	8 (12)			9 (6)	0.2 (−0.5 to 1)	0.376
**Week 24**	5 (8)	0.1 (−0.5 to 0.7)	0.753	3 (5)	−0.5 (−1.3 to 0.4)	0.733
**CMJ relative power (w/BW)**						
**Week 10**	42 (6)			44 (6)	0.3 (−0.4 to 1.1)	0.349
**Week 24**	38 (4)	0.4 (−0.3 to 1.1)	0.310	43 (6)	0.9 (0.1 to 1.6)	0.089
**RSI (m/s)**						
**Week 10**	0.4 (0.1)			0.4 (0.1)	0.3 (−0.4 to 1)	0.534
**Week 24**	0.3 (0.1)	0.7 (0 to 1.4)	0.107	0.4 (0.1)	1.1 (0.3 to 1.8)	0.010
**Nordic left (N/BW)**						
**Week 10**	4.9 (0.9)			4.9 (1)	0 (−0.7 to 0.7)	0.887
**Week 24**	4.8 (0.5)	0.3 (−0.4 to 0.9)	0.345	4.7 (0.6)	−0.2 (−0.9 to 0.6)	0.802
**Nordic right (N/BW)**						
**Week 10**	4.6 (0.8)			4.7 (0.5)	0.2 (−0.5 to 0.9)	0.798
**Week 24**	4.6 (0.8)	0.2 (−0.5 to 0.8)	0.753	4.5 (0.5)	−0.2 (−1 to 0.6)	0.541

*s*Fer—serum ferritin; TD >50%—total distance covered greater than 50% of maximal velocity; TD > 70%—total distance covered greater than 70% of maximal velocity; TD > 80%—total distance covered greater than 80% of maximal velocity; TD > 90%—total distance covered greater than 90% of maximal velocity; Nordic left—Nordic hamstring curl left side; Nordic right—Nordic hamstring curl right side.

**Table 4:** Relative predictions for physical performance measures from the ReML analysis based on the interactions of fixed (sFer, iron group, week, and position) and random (athlete) effects.

	Performance Measure (Response Measure)							
	TD 50%+ (m)	TD 70%+ (m)	TD 80%+ (m)	TD 90%+ (m)	CMJ Relative Power (w/BW)	RSI S (m/s)	Nordic Left (N/BW)	Nordic Right (N/BW)
**Intercept**	1447 (1142 to 1751)	80 (−3 to 162)	32 (7 to 58)	11 (−3 to 26)	45 (42 to 48)	0.4 (0.4 to 0.5)	4.7 (4.1 to 5.3)	4.9 (4.5 to 5.3)
**sFer**	−1 (−6 to 4)	1 (0 to 2)	0 (0 to 1)	0 (0 to 0)	0.0 (−0.1 to 0.0)	0 (0 to 0)	0 (0 to 0)	0 (0 to 0)
**FeUP**	−62 (−364 to 240)	−14 (−95 to 67)	2 (−23 to 28)	6 (−9 to 20)	−2.0 (−5.1 to 1.2)	−0.1 (−0.1 to 0)	0.3 (−0.4 to 1)	−0.2 (−0.6 to 0.3)
**Mid**	166 (−160 to 498)	31 (−57 to 122)	6 (−21 to 32)	−2 (−18 to 13)				
**Week 1**	0	0	0	0	0	0	0	0
**Week 10**	−374 (−724 to −29)	112 (19 to 205)	9 (−21 to 39)	11 (−6 to 28)	−0.9 (−3.6 to 1.8)	0.0 (−0.1 to 0.0)	0.0 (−0.6 to 0.7)	−0.1 (−0.5 to 0.4)
**Week 24**	−378 (−702 to −56)	45 (−41 to 133)	−12 (−41 to 17)	−14 (−30 to 2)	−3.0 (−5.9 to −0.2)	0.0 (−0.1 to 0.0)	0.1 (−0.6 to 0.8)	0.0 (−0.5 to 0.4)
**sFer:Mid**	1 (−6 to 8)	0 (−2 to 2)	0 (−1 to 1)	0 (0 to 1)				
**sFer:FeUP**	3 (−4 to 10)	0 (−1 to 2)	0 (−1 to 1)	0 (0 to 0)	0.0 (−0.1 to 0.1)	0 (0 to 0)	0 (0 to 0)	0 (0 to 0)
**sFer:Week 10**	−3 (−12 to 5)	−2 (−4 to 0)	0 (−1 to 1)	0 (−1 to 0)	0.0 (0.0 to 0.1)	0 (0 to 0)	0 (0 to 0)	0 (0 to 0)
**sFer:Week 24**	1 (−7 to 9)	−1 (−3 to 1)	0 (−1 to 1)	0 (0 to 1)	0.0 (0.0 to 0.1)	0 (0 to 0)	0 (0 to 0)	0 (0 to 0)
**R2m/R2c**	0.4/0.7	0.1/0.5	0.1/0.3	0.2/0.5	0.0/0.9	0.0/0.8	0.0/0.6	0.0/0.7

sFer—serum ferritin; Mid—midfielders; TD >50%—total distance covered greater than 50% of maximal velocity; TD > 70%—total distance covered greater than 70% of maximal velocity; TD > 80%—total distance covered greater than 80% of maximal velocity; TD > 90%—total distance covered greater than 90% of maximal velocity; Nordic left—Nordic hamstring curl left side; Nordic right—Nordic hamstring curl right side. Week 1 is used as the reference week. R^2^m indicates the variance explained by the fixed effects. R^2^c indicates the variance explained by both the fixed and random effects.

## Data Availability

Data supporting the study’s findings are available from the corresponding author upon reasonable request, but are not publicly shared due to ethical and privacy considerations.

## Supplementary Materials

The following supporting information can be downloaded at https://www.mdpi.com/article/10.3390/sports13050136/s1: Table S1. Physical performance measures recorded throughout the season for both players who received an iron infusion with differences in each measure expressed as a percentage. Table S2. Physical performance measures (mean ± SD) recorded during week 10 and week 24 between players with heightened iron status (FeUP) and non-supplemented (Ctrl) AFLW players with effect size comparisons. Table S3. Relative predictions for external load measures total distance and PlayerLoad from the ReML analysis based on the interactions of fixed (sFer, iron group, week, and position) and random (athlete) effects. Table S4. Relative predictions for all external load measures from the ReML analysis based on the interactions of fixed (log(sFer), iron group, week, and position) and random (athlete) effects. Table S5. Relative predictions for strength and power measures hip abduction, hip adduction, and isometric 30˚ hamstring hold from the ReML analysis based on the interactions of fixed (sFer, iron group, and week) and random (athlete) effects. Table S6. Relative predictions for all strength and power measures from the ReML analysis based on the interactions of fixed (log(sFer), iron group, and week) and random (athlete) effects. Figure S1. Distribution plot of the relative total distance and PlayerLoad per hour for each individual separated by group (Ctrl and FeUP) and week. The FeUP group (purple) represents those players with heightened iron status between weeks 10 and 24. Unfilled purple triangles indicate the two players who received an iron infusion after week 1, while filled purple circles represent all other FeUP players. The Ctrl group (green) represents all non-supplemented players between weeks 10 and 24. Figure S2. Distribution plot of the relative hip abduction, hip adduction, and isometric 30˚ hamstring force output for each individual separated by group (Ctrl and FeUP) and week. The FeUP group (purple) represents those players with heightened iron status between weeks 10 and 24. Unfilled purple triangles indicate the two players who received an iron infusion after week 1, while filled purple circles represent all other FeUP players. The Ctrl group (green) represents all non-supplemented players between weeks 10 and 24. Figure S3. Scatterplot of the relative total distance and PlayerLoad recorded per hour for each individual athlete plotted against serum ferritin (sFer), separated by week. The purple line represents the predicted total distance and PlayerLoad covered by the players with heightened iron status between weeks 10 and 24 (FeUP) per hour, with 95% CIs in purple. Unfilled purple triangles indicate the two players who received an iron infusion after week 1, while filled purple circles represent all other FeUP players. The green line represents the predicted total distance and PlayerLoad covered by the non-supplemented players (Ctrl) per hour, with 95% CIs in green. The red line indicates an sFer of 40 µg/L, the diagnostic cut off used in the current study to categorise iron deficiency. Figure S4. Scatterplot of the relative hip abduction, hip adduction, and isometric 30˚ hamstring force output for each individual athlete plotted against serum ferritin (sFer), separated by week. The purple line represents the predicted total distance and PlayerLoad covered by the players with heightened iron status between weeks 10 and 24 (FeUP) per hour, with 95% CIs in purple. Unfilled purple triangles indicate the two players who received an iron infusion after week 1, while filled purple circles represent all other FeUP players. The green line represents the predicted total distance and PlayerLoad covered by the non-supplemented players (Ctrl) per hour, with 95% CIs in green. The red line indicates an sFer of 40 µg/L, the diagnostic cut off used in the current study to categorise iron deficiency.

## Abbreviations

The following abbreviations are used in this manuscript:

FeUP	Heightened iron status group
Ctrl	Non-supplemented group
sFer	Serum ferritin
ID	Iron deficient
AFLW	Australian Football League Women’s
IDNA-1	Stage one iron deficiency non-anemia
IDNA-2	Stage two iron deficiency non-anemia
IDA-3	Stage three iron deficiency anemia
Hb	Hemoglobin concentration
TD	Total distance
RSI	Reactive strength index
CMJ	Counter-movement jump
CI	Confidence interval
REML	Restricted maximum likelihood
FWD/BACK/KEY	Forward, back, or key position player
MID	Midfielder
