# Supplementary material for: Putting the Fe into Female Athletes: Insights into Heightened Iron Status and Women’s Australian Football Performance—A Case Study
Source: Sports (Basel). 2025 Apr 29;13(5):136. doi: 10.3390/sports13050136 (PMC12115492; doi:10.3390/sports13050136)
Supplement: Supplementary file 1 [file sports-13-00136-s001.zip › sports-3569814-supplementary.pdf]

Table S1. Physical performance measures recorded throughout the season for both players who received an iron infusion with differences in each measure expressed as a percentage.

Performance measure	Athlete	Week 1	Week 10	Week 24	Difference in measure (%)		
					Week 1:Week 10	Week 1:Week 24	Week 10:Week 24
Total distance (m)	Player 1	5265	5415	5569	3%	6%	3%
	Player 2	5621	-	5723	-	2%	-
PlayerLoad (a.u.)	Player 1	548	605	610	10%	11%	1%
	Player 2	468	-	482	-	3%	-
Abd. left (N/BW)	Player 1	-	2.6	2.5	-	-	-4%
	Player 2	-	2.1	2.4	-	-	13%
Abd. right (N/BW)	Player 1	-	2.6	2.4	-	-	-9%
	Player 2	-	2.2	2.2	-	-	-3%
Add. left (N/BW)	Player 1	-	2.8	2.9	-	-	4%
	Player 2	-	2.0	2.3	-	-	16%
Add. right (N/BW)	Player 1	-	2.9	2.9	-	-	0%
	Player 2	-	1.9	2.0	-	-	3%
Iso30 left (N/BW)	Player 1	-	6.2	4.9	-	-	-21%
	Player 2	-	4.8	4.8	-	-	0%
Iso30 right (N/BW)	Player 1	-	6.2	5.1	-	-	-18%
	Player 2	-	4.7	5.0	-	-	6%

Abd. left hip abduction left side, Abd. right hip abduction right side, Add. left hip adduction left side, Add. right hip adduction right side, Iso30 left Isometric 30˚ hamstring hold left side, Iso30 right Isometric 30˚ hamstring hold right side.

Table S2. Physical performance measures (mean ± SD) recorded during week 10 and week 24 between players with heightened iron status (FeUP) and non-supplemented (Ctrl) AFLW players with effect size comparisons.

Iron measure	FeUP	Cohen's d (90% CIs)	p value	Ctrl	Cohen's d (90% CIs)	p value
	Mean (SD)	FeUP Week 10:Week 24		Mean (SD)	Ctrl:FeUP	
Total distance (m)						
Week 10	5248 (231)			5279 (302)	0.1 (-0.6 to 0.9)	.871
Week 24	5622 (511)	-0.6 (-1.3 to 0)	.123	5782 (355)	0.4 (-0.4 to 1.2)	.850
PlayerLoad (a.u.)						
Week 10	559 (42)			551 (59)	-0.2 (-0.9 to 0.6)	.413
Week 24	603 (79)	-0.3 (-0.9 to 0.3)	.575	607 (78)	0.1 (-0.7 to 0.9)	.677
Abd. left (N/BW)						
Week 10	2.2 (0.2)			2.3 (0.4)	0 (-0.7 to 0.8)	1.00
Week 24	2.3 (0.4)	0 (-0.6 to 0.6)	.528	2.3 (0.3)	0.2 (-0.5 to 0.9)	.787
Abd. right (N/BW)						
Week 10	2.3 (0.3)			2.2 (0.3)	-0.2 (-0.9 to 0.6)	.664
Week 24	2.3 (0.5)	0.1 (-0.5 to 0.7)	.734	2.3 (0.3)	0.1 (-0.6 to 0.8)	.928
Add. left (N/BW)						
Week 10	2.2 (0.5)			2.1 (0.4)	-0.1 (-0.8 to 0.6)	.977
Week 24	2.3 (0.5)	-0.4 (-1 to 0.2)	.344	2.2 (0.5)	-0.2 (-0.9 to 0.5)	.881
Add. right (N/BW)						
Week 10	2.2 (0.5)			2.1 (0.4)	-0.3 (-1 to 0.4)	.842
Week 24	2.4 (0.5)	-0.4 (-1 to 0.2)	.397	2.2 (0.4)	-0.5 (-1.2 to 0.3)	.417
Iso30 left (N/BW)						
Week 10	4.8 (1.4)			4.6 (1.2)	-0.2 (-0.9 to 0.6)	.798
Week 24	4.5 (1)	0.1 (-0.5 to 0.7)	.446	4.5 (1)	0 (-0.8 to 0.7)	.974
Iso30 right (N/BW)						
Week 10	4.7 (1.2)			4.5 (1)	-0.2 (-1 to 0.5)	.588
Week 24	4.4 (0.8)	0.1 (-0.5 to 0.7)	.672	4.2 (0.8)	-0.3 (-1.1 to 0.4)	.341

Abd. left hip abduction left side, Abd. right hip abduction right side, Add. left hip adduction left side, Add. right hip adduction right side, Iso30 left Isometric 30˚ hamstring hold left side, Iso30 right Isometric 30˚ hamstring hold right side.

Table S3. Relative predictions for external load measures total distance and PlayerLoad from the ReML analysis based on the interactions of fixed (sFer, iron group, week, and position) and random (athlete) effects.

	External load measure (response measure)	
	Total distance (m)	PlayerLoad (a.u.)
Intercept	4956 (4580 to 5336)	477 (415 to 537)
sFer	-3 (-9 to 4)	0 (-1 to 1)
FeUP	-377 (-741 to -14)	-42 (-100 to 17)
Mid	347 (-43 to 745)	57 (-9 to 127)
Week 1	0	0
Week 10	397 (-38 to 821)	85 (19 to 150)
Week 24	720 (314 to 1119)	147 (88 to 207)
log(sFer):Mid	-5 (-14 to 4)	0 (-2 to 1)
log(sFer):FeUP	9 (0 to 17)	1 (0 to 2)
log(sFer):Week 10	-1 (-11 to 9)	-1 (-2 to 1)
log(sFer):Week 24	2 (-8 to 12)	-1 (-3 to 0)
R^2m/ R^2c	0.6/0.7	0.4/0.8

sFer serum ferritin, *FeUP* players with heightened iron status between weeks 10 and 24, *Mid* Midfielders.

Note: Week 1 is used as the reference week. R^2m indicates the variance explained by the fixed effects. R2c indicates the variance explained by both the fixed and random effects.

Table S4. Relative predictions for all external load measures from the ReML analysis based on the interactions of fixed (log(sFer), iron group, week, and position) and random (athlete) effects.

	External load measure (response measure)					
	TD >50% (m)	TD >70% (m)	TD >80% (m)	TD >90% (m)	Total distance (m)	PlayerLoad (a.u.)
Intercept	1604 (628 to 2585)	-74 (-337 to 190)	8 (-73 to 90)	-2 (-24 to 20)	5190 (3984 to 6413)	489 (296 to 680)
sFer	-52 (-302 to 197)	53 (-14 to 120)	9 (-12 to 29)	1 (-4 to 7)	-98 (-410 to 210)	-4 (-52 to 45)
FeUP	-402 (-1337 to 536)	-76 (-323 to 171)	26 (-52 to 104)	19 (-3 to 41)	-989 (-2099 to 122)	-122 (-302 to 57)
Mid	121 (-919 to 1167)	135 (-150 to 426)	37 (-45 to 120)	-14 (-37 to 9)	1018 (-269 to 2317)	83 (-125 to 296)
Week 1	0	0	0	0	0	0
Week 10	-84 (-1187 to 1003)	338 (54 to 623)	39 (-56 to 132)	26 (-2 to 52)	452 (-873 to 1758)	175 (-30 to 379)
Week 24	-725 (-1794 to 330)	71 (-205 to 351)	-17 (-111 to 77)	-19 (-47 to 7)	-4 (-1297 to 1276)	196 (2 to 390)
log(sFer):Mid	23 (-261 to 306)	-24 (-103 to 53)	-10 (-32 to 13)	4 (-2 to 11)	-243 (-596 to 108)	-9 (-66 to 47)
log(sFer):FeUP	128 (-136 to 392)	21 (-48 to 90)	-7 (-29 to 14)	-5 (-12 to 1)	260 (-51 to 571)	33 (-17 to 84)
log(sFer):Week 10	-118 (-419 to 188)	-85 (-164 to -7)	-10 (-36 to 16)	-6 (-13 to 2)	-23 (-385 to 342)	-35 (-91 to 22)
log(sFer):Week 24	108 (-187 to 406)	-14 (-93 to 63)	2 (-24 to 28)	6 (-1 to 14)	232 (-126 to 591)	-26 (-81 to 28)
R^2m/ R^2c	0.4/0.7	0.1/0.6	0.2/0.3	0.3/0.4	0.6/0.8	0.4/0.8

sFer serum ferritin, FeUP players with heightened iron status between weeks 10 and 24, Mid Midfielders, TD50%+ Total distance covered greater than 50% of maximal velocity, TD70%+ Total distance covered greater than 70% of maximal velocity, TD80%+ Total distance covered greater than 80% of maximal velocity, TD90%+ Total distance covered greater than 90% of maximal velocity, Nordic left Nordic hamstring curl left side, Nordic right Nordic hamstring curl right side.

Note: Week 1 is used as the reference week. R^2m indicates the variance explained by the fixed effects. R2c indicates the variance explained by both the fixed and random effects.

Table S5. Relative predictions for strength and power measures hip abduction, hip adduction, and isometric 30˚ hamstring hold from the ReML analysis based on the interactions of fixed (sFer, iron group, and week) and random (athlete) effects.

	Strength and power measure (response measure)					
	Abd. left (N/BW)	Abd. right (N/BW)	Add. left (N/BW)	Add. Right (N/BW)	Iso30 left (N/BW)	Iso30 right (N/BW)
Intercept	2.2<br>(1.8 to 2.5)	2<br>(1.6 to 2.4)	2.1<br>(1.5 to 2.6)	2<br>(1.5 to 2.5)	4.8<br>(3.8 to 5.8)	4.7<br>(3.7 to 5.7)
sFer	0<br>(0 to 0)	0<br>(0 to 0)	0<br>(0 to 0)	0<br>(0 to 0)	0<br>(0 to 0)	0<br>(0 to 0)
FeUP	0<br>(-0.4 to 0.5)	0.2<br>(-0.3 to 0.6)	0<br>(-0.6 to 0.6)	0.1<br>(-0.4 to 0.7)	0<br>(-1.4 to 1.3)	0.1<br>(-1.1 to 1.3)
Week 10	0	0	0	0	0	0
Week 24	0.1<br>(-0.1 to 0.3)	0<br>(-0.2 to 0.3)	0<br>(-0.2 to 0.4)	0.1<br>(-0.2 to 0.4)	0.1<br>(-0.3 to 0.6)	-0.1<br>(-0.5 to 0.4)
log(sFer):FeUP	0<br>(0 to 0)	0<br>(0 to 0)	0<br>(0 to 0)	0<br>(0 to 0)	0<br>(0 to 0)	0<br>(0 to 0)
log(sFer):Week 24	0<br>(0 to 0)	0<br>(0 to 0)	0<br>(0 to 0)	0<br>(0 to 0)	0<br>(0 to 0)	0<br>(0 to 0)
R^2m/ R^2c	0.0/0.7	0.0/0.7	0.1/0.6	0.1/0.6	0.0/0.9	0.1/0.8

sFer serum ferritin, FeUP players with heightened iron status between weeks 10 and 24, Abd. left hip abduction left side, Abd. right hip abduction right side, Add. left hip adduction left side, Add. right hip adduction right side, Iso30 left Isometric 30˚ hamstring hold left side, Iso30 right Isometric 30˚ hamstring hold right side.

Note: Week 10 is used as the reference week. R^2m indicates the variance explained by the fixed effects. R2c indicates the variance explained by both the fixed and random effects.

Table S6. Relative predictions for all strength and power measures from the ReML analysis based on the interactions of fixed (log(sFer), iron group, and week) and random (athlete) effects.

	Strength and power measure (response measure)									
	CMJ relative power (w/BW)	RSI (m/s)	Nordic left (N/BW)	Nordic right (N/BW)	Abd. left (N/BW)	Abd. right (N/BW)	Add. left (N/BW)	Add. right (N/BW)	Iso30 left (N/BW)	Iso30 right (N/BW)
Intercept	46 (38 to 54)	0.5 (0.3 to 0.6)	4.4 (2.6 to 6.3)	5.5 (4.3 to 6.8)	1.9 (0.7 to 3)	1.5 (0.3 to 2.8)	2.1 (0.5 to 3.7)	2 (0.4 to 3.5)	5.1 (1.9 to 8.4)	5.2 (2.2 to 8.3)
sFer	-0.6 (-2.8 to 1.5)	0.0 (-0.1 to 0.0)	0.1 (-0.4 to 0.6)	-0.2 (-0.6 to 0.1)	0.1 (-0.2 to 0.4)	0.2 (-0.1 to 0.5)	0 (-0.4 to 0.4)	0 (-0.4 to 0.5)	-0.2 (-1.1 to 0.7)	-0.2 (-1.1 to 0.6)
FeUP	-0.3 (-9.1 to 8.6)	-0.1 (-0.2 to 0.1)	1.3 (-0.7 to 3.3)	-0.2 (-1.5 to 1.2)	0.3 (-1 to 1.6)	0.6 (-0.9 to 2)	-0.3 (-2.1 to 1.6)	0.1 (-1.8 to 1.8)	0.8 (-3.5 to 4.9)	1 (-2.8 to 4.9)
Week 1	0	0	0	0						
Week 10	-0.9 (-9.8 to 8.0)	0.0 (-0.1 to 0.2)	-0.2 (-2.3 to 1.9)	-0.2 (-1.6 to 1.3)	0	0	0	0	0	0
Week 24	-7.5 (-17 to 2.4)	0.0 (-0.2 to 0.2)	-0.1 (-2.5 to 2.2)	-0.5 (-2 to 1.1)	0.2 (-0.5 to 1)	0.1 (-0.7 to 1)	-0.4 (-1.5 to 0.8)	0 (-1.1 to 1)	1.3 (-0.4 to 3)	0.7 (-1.1 to 2.4)
log(sFer):FeUP	-0.5 (-2.9 to 1.9)	0.0 (0.0 to 0.1)	-0.3 (-0.9 to 0.2)	0 (-0.3 to 0.4)	-0.1 (-0.5 to 0.3)	-0.2 (-0.6 to 0.2)	0.1 (-0.4 to 0.6)	0 (-0.5 to 0.5)	-0.1 (-1.2 to 1.1)	-0.1 (-1.2 to 0.9)
log(sFer):Week 10	0.1 (-2.3 to 2.6)	0.0 (-0.1 to 0.0)	0.1 (-0.5 to 0.7)	0 (-0.4 to 0.4)						
log(sFer):Week 24	1.7 (-1.0 to 4.4)	0.0 (0.0 to 0.1)	0 (-0.6 to 0.7)	0.1 (-0.3 to 0.5)	0 (-0.2 to 0.2)	0 (-0.3 to 0.2)	0.1 (-0.2 to 0.4)	0 (-0.2 to 0.3)	-0.4 (-0.8 to 0.1)	-0.2 (-0.7 to 0.2)
R^2m/ R^2c	0.0/0.9	0.0/0.8	0.0s/0.6	0.0/0.7	0.0/0.7	0.0/0.7	0.1/0.6	0.1/0.6	0.1/0.9	0.1/0.8

sFer serum ferritin, FeUP players with heightened iron status between weeks 10 and 24, CMJ counter-movement jump, RSI reactive strength index, Nordic left Nordic hamstring curl left side, Nordic right Nordic hamstring curl right side, Abd. left hip abduction left side, Abd. right hip abduction right side, Add. left hip adduction left side, Add. right hip adduction right side, Iso30 left Isometric 30˚ hamstring hold left side, Iso30 right Isometric 30˚ hamstring hold right side.

Note: Week 1 is used as the reference week for CMJ relative power, RSI, and Nordics. Week 10 is used as the reference week for all other measures.. R^2m indicates the variance explained by the fixed effects. R2c indicates the variance explained by both the fixed and random effects.

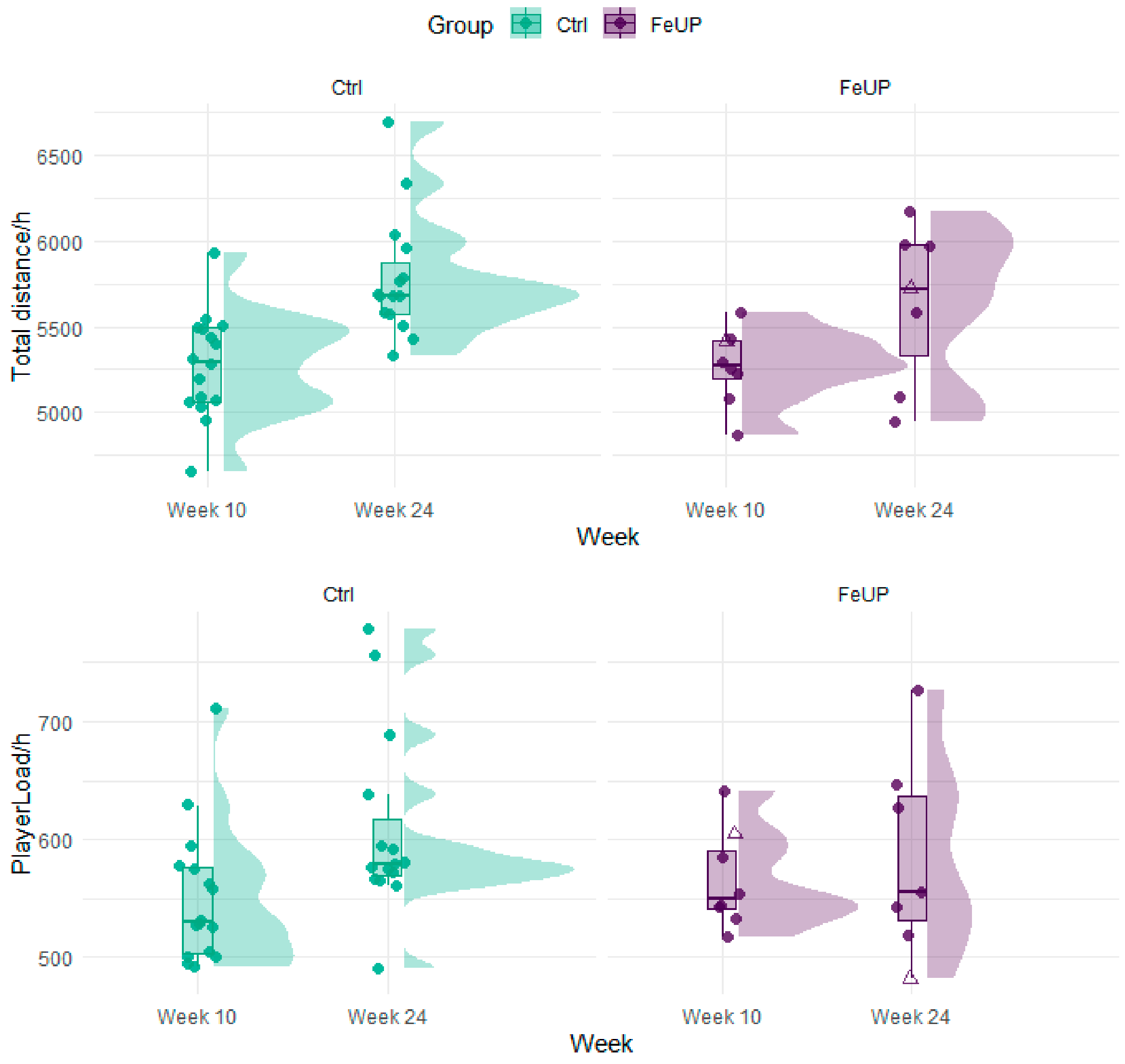

Figure S1. Distribution plot of the relative total distance and PlayerLoad per hour for each individual separated by group (Ctrl and FeUP) and week. The FeUP group (purple) represents those players with heightened iron status between weeks 10 and 24. Unfilled purple triangles indicate the two players who received an iron infusion after week 1, while filled purple circles represent all other FeUP players. The Ctrl group (green) represents all non-supplemented players between weeks 10 and 24.

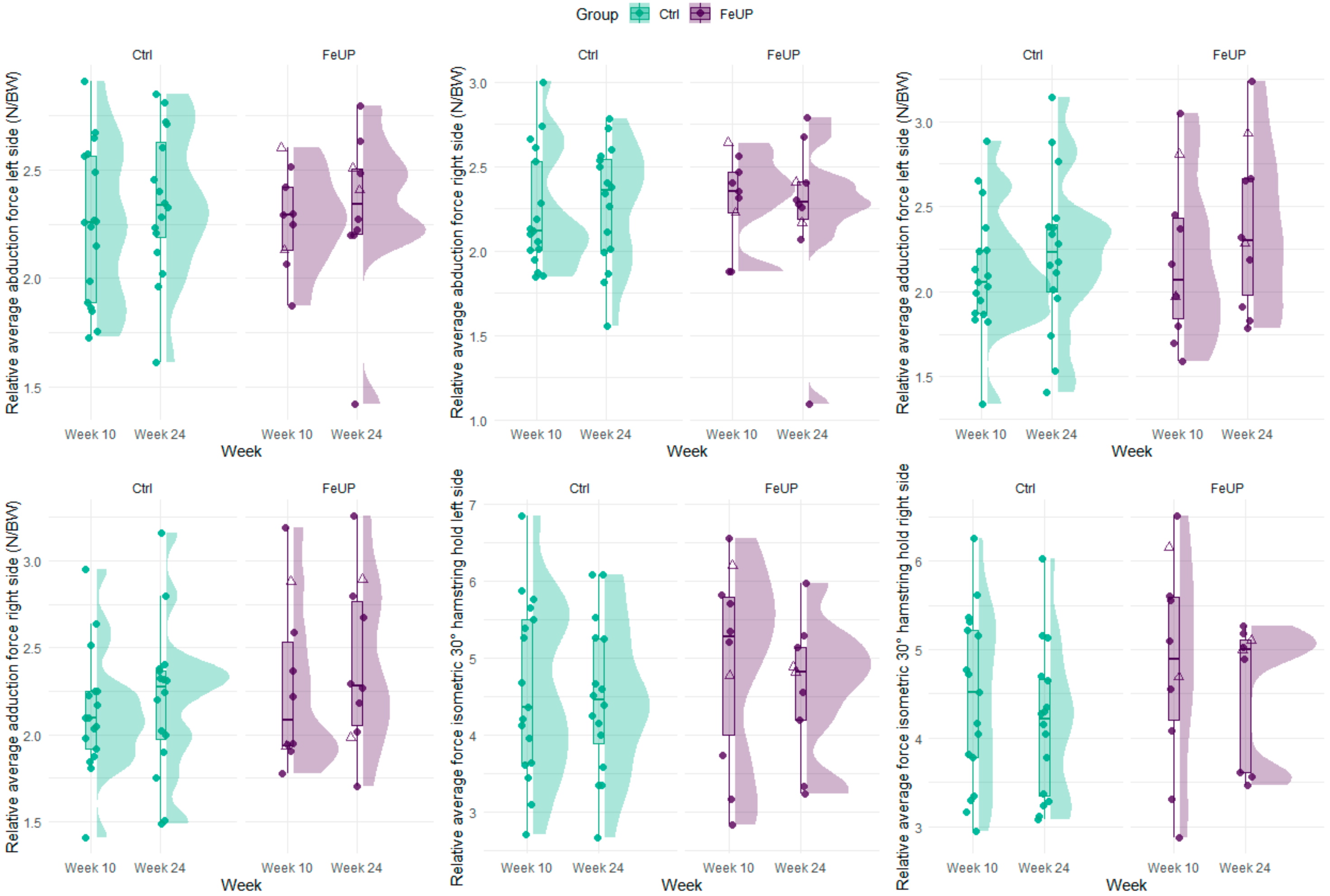

Figure S2. Distribution plot of the relative hip abduction, hip adduction, and isometric 30˚ hamstring force output for each individual separated by group (Ctrl and FeUP) and week. The FeUP group (purple) represents those players with heightened iron status between weeks 10 and 24. Unfilled purple triangles indicate the two players who received an iron infusion after week 1, while filled purple circles represent all other FeUP players. The Ctrl group (green) represents all non-supplemented players between weeks 10 and 24.

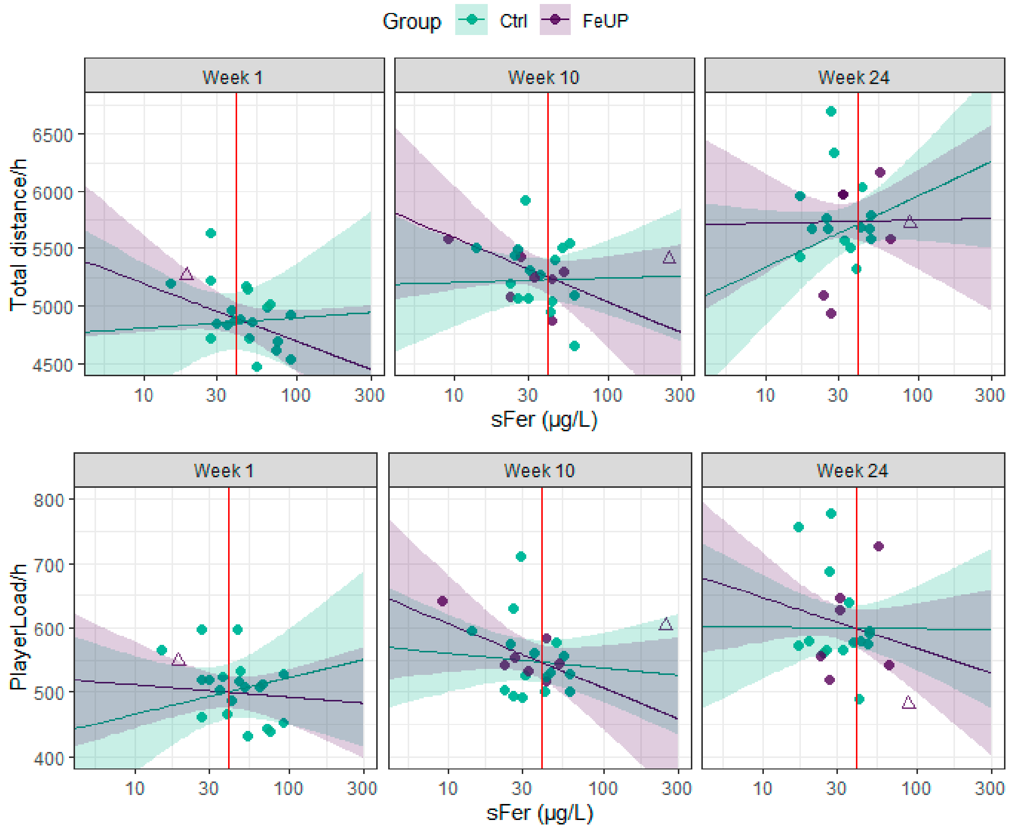

Figure S3. Scatterplot of the relative total distance and PlayerLoad recorded per hour for each individual athlete plotted against serum ferritin (sFer), separated by week. The purple line represents the predicted total distance and PlayerLoad covered by the players with heightened iron status between weeks 10 and 24 (FeUP) per hour, with 95% CIs in purple. Unfilled purple triangles indicate the two players who received an iron infusion after week 1, while filled purple circles represent all other FeUP players. The green line represents the predicted total distance and PlayerLoad covered by the non-supplemented players (Ctrl) per hour, with 95% CIs in green. The red line indicates an sFer of 40 µg/L, the diagnostic cut off used in the current study to categorise iron deficiency.

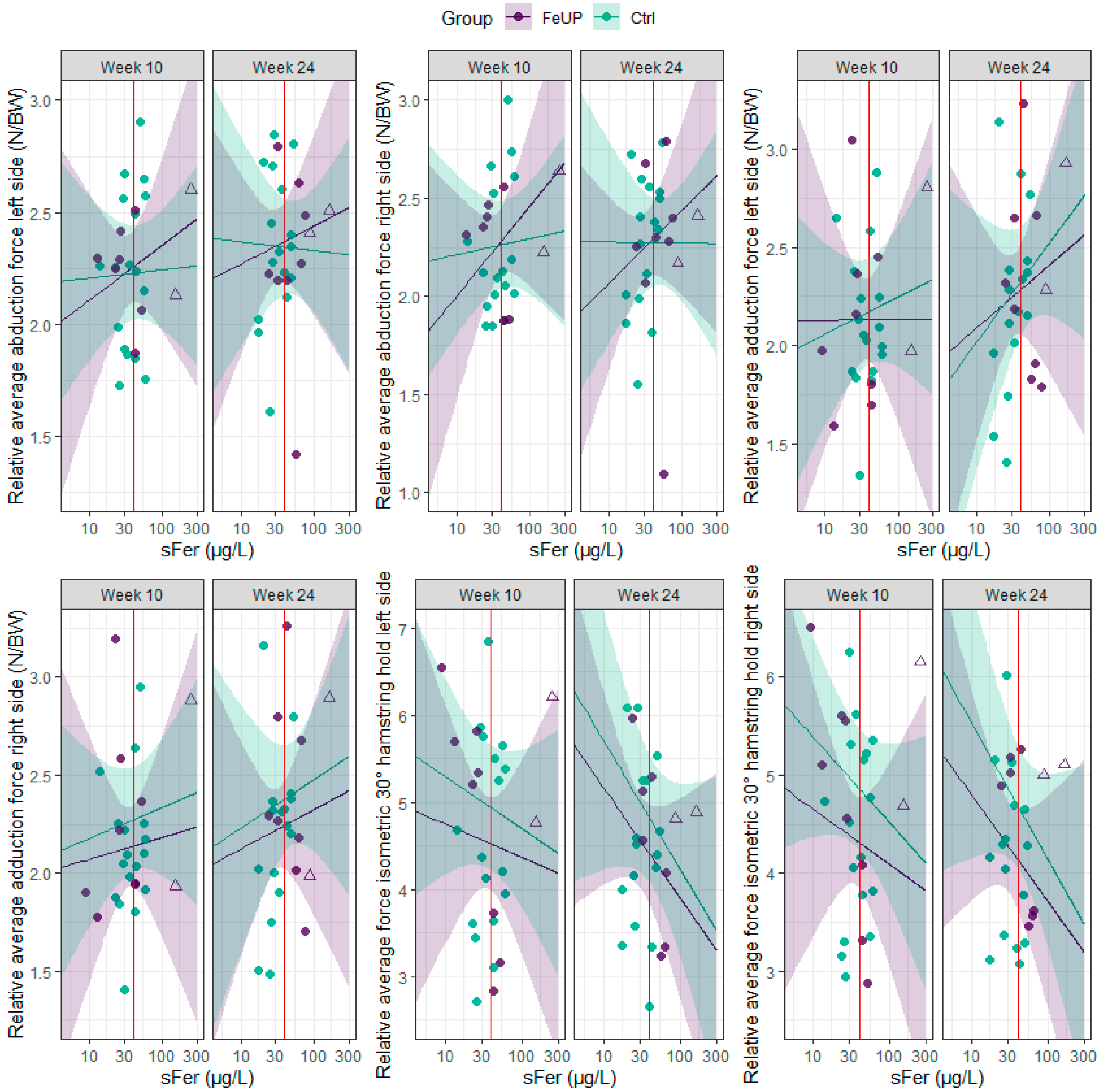

Figure S4. Scatterplot of the relative hip abduction, hip adduction, and isometric 30˚ hamstring force output for each individual athlete plotted against serum ferritin (sFer), separated by week. The purple line represents the predicted total distance and PlayerLoad covered by the players with heightened iron status between weeks 10 and 24 (FeUP) per hour, with 95% CIs in purple. Unfilled purple triangles indicate the two players who received an iron infusion after week 1, while filled purple circles represent all other FeUP players. The green line represents the predicted total distance and PlayerLoad covered by the non-supplemented players (Ctrl) per hour, with 95% CIs in green. The red line indicates an sFer of 40 µg/L, the diagnostic cut off used in the current study to categorise iron deficiency.
